# Consequences of Recent Crises on the FLW Consumer Behaviour: A National Wide Representative Research—The Case of Romania

**DOI:** 10.3390/foods12101973

**Published:** 2023-05-12

**Authors:** Cristina Bianca Pocol, Antonio Amuza, Maria-Georgeta Moldovan, Liana Stanca, Dan-Cristian Dabija

**Affiliations:** 1 Department of Animal Production and Food Safety, University of Agricultural Sciences and Veterinary Medicine of Cluj-Napoca, 400372 Cluj-Napoca, Romania; cristina.pocol@usamvcluj.ro; 2 Department of Sociology and Social Work, University of Bucharest, 030018 Bucharest, Romania; amuza.antonio@gmail.com; 3 Department of Business Informatics, Faculty of Economics and Business Administration, Babeș-Bolyai University, 400591 Cluj-Napoca, Romania; liana.stanca@ubbcluj.ro; 4 Department of Marketing, Faculty of Economics and Business Administration, Babeș-Bolyai University, 400591 Cluj-Napoca, Romania

**Keywords:** food loss and waste (FLW), food consumption behaviour, agri-food chain, Romanian consumers, cluster analysis, typologies, emerging market

## Abstract

Research on food loss and waste (FLW) is quite limited in emerging countries, such as Romania, as the phenomenon, its consequences, and implications are not yet properly understood by both policy makers and consumers. Therefore, the aim of this paper is to conduct representative research in Romania to identify the main clusters of consumers depending on their food waste behaviour. By means of cluster analysis, we highlight the main consumer typologies in Romania, regarding their food waste behaviour. The main findings reveal the presence of three distinct segments of consumer typologies based on their food waste behaviour, including low-income young wasters, conscious middle-age wasters, and well-educated mature non-wasters. This study highlights the need for targeted interventions that consider the unique characteristics and behaviours of each segment to effectively reduce FLW at the household level. Overall, this paper provides important insights for academia and for policymakers in the field of FLW management. The food loss and waste behaviour has significant economic, social, and environmental impacts, and reducing it requires a common effort from all stakeholders. Reducing food waste presents challenges, but also presents an opportunity to improve economic, social, and environmental outcomes.

## 1. Introduction

One-third of global food production is lost or wasted along the agri-food chain, so food loss and waste is increasingly becoming a major concern for worldwide policy makers and researchers [1,2,3,4], and the investigation of the possible causes of this phenomenon is being conducted through a multidisciplinary approach [5,6,7,8]. Identifying solutions, which could lead to the reduction and alleviation of the FLW problem, remains a global priority [1,9,10]. In this sense, it would also meet one of the specific objectives of the United Nations Sustainable Development Strategy, namely, to reduce food loss at the household and retailer level by 2030, but also to considerably diminish food waste in production and transformation processes and supply chains [11,12].

The United Nations estimate through the Food and Agricultural Organization [11,12] that 14% of the entire food which is produced yearly for mankind consumption is either lost or wasted globally along agri-food chains, from farm to fork. The highest loss occurs in Sub-Saharan Africa (21.4%), whereas the lowest loss has been reported in Europe and North America (9.9%) [11]. Food waste at the retail and final consumer levels was estimated at 931 million tonnes in 2019, representing 17% of the total global food production, with 11% of waste at the household level [13]. This worrying trend is also confirmed by a large number of recent studies [14,15,16,17,18,19], attesting that waste at the household level exerts significant negative economic, social, and environmental consequences as follows: the over-production of food leads to the over-use of already limited natural resources (such as energy, water, fossil fuels, and land) and global warming (increase in greenhouse emissions); over-purchase due to the lack of adjustment between necessity and consumption affects the solidity of the families’ economic model (many of them, already vulnerable and financially struggling, could use the money wisely on other priorities); over-depositing of the mountains of uneaten or unsold food products require supplementary resources (means for management, extra-handling and reuse in labour, oil/fuel, warehouses); and lastly, we cannot speak of food waste without tackling ethics (knowing the hunger-affected areas of the world, households should be more aware of the need for social responsibility to which they also need to fall in line) [20,21,22,23].

While it is possible to find studies on the FLW phenomenon in countries of the European Union [24,25,26], in Romania, research is limited in terms of number, representation of the samples, as well as the strategies identified and implemented [27,28,29,30]. Furthermore, many FLW-related studies at the international level, which are based on secondary data, show that there is a direct proportional increase in household food waste per capita and GDP per capita [1]. Therefore, the literature is calling for further and more consistent primary data-based research, especially for emerging economies [1].

The recent literature approaches the FLW phenomenon with the help of a methodological mix, using research instruments such as questionnaires and in-depth interview guides, and using the diary method and/or other direct measurements less frequently. Quite often, research instruments such as questionnaires are distributed through social networks [30,31,32,33], with the data being collected either before or during the recent COVID-19 pandemic [28,31]. Such studies have quite often a limited size and sample representativeness [27,31,34], only being implemented on the narrowed survey areas [30,34,35].

The lack of statistical data on the FLW situation in Romania [36,37], as well as the deficiencies highlighted in the literature, combined with the current geo-political and socio-economic context (systemic and multi-sectoral crisis in the military, economic, social, health, energy, and food sectors), have all justified national research on anti-waste behaviour at the household level.

Therefore, the aim of this paper is to implement representative research in terms of household size, age, and gender to pinpoint the main food waste consumer typologies. In this regard, the authors relied on a cluster analysis, because it represents an exploratory method that consists of demonstrating the presence of homogeneous structures of component parts [38] and of taking several predefined steps, namely, choosing, and standardising variables; finding a similarity index; applying the clustering approach; evaluating the typologies; and identifying the appropriate number of segments and naming them. The novelty of the approach is twofold; on the one hand, it is based on representative nationwide research aimed at delimitating consumer clusters, while on the other hand, it offers a new perspective on FLW behaviour based on consumers’ pandemic experience, as well as the recent regional developments of the armed conflict between Ukraine and Russia.

The structure of this paper is as follows: Section 2 contains the materials and methods where the authors present the literature review of the investigated phenomenon, but also the research methodology. Section 3 contains the results analysis and the discussions, while the paper ends with relevant conclusions, containing the theoretical and managerial implications, along with the limitations and future research directions.

## 2. Materials and Methods

### 2.1. Literature Review

#### 2.1.1. Recent FLW Developments

The literature highlights the relevance of researching the food loss and waste phenomenon, pinpointing the necessity to identify proper methods and tools to reduce its impact within agri-food supply chains. Furthermore, the recent literature [31,32,38,39] also shows FLW causes, internal/external determinants, and opportunities for recovery through recycling or reuse of food waste. Identifying and implementing strategies to mitigate or reduce the FLW phenomenon in the medium and long term is a major challenge facing the international community to ensure sufficient safe food for the soon-to-be 9 billion people on Earth by 2050 [11,12,40].

Unpredictable situations, such as health crises [38,41], natural disasters due to climate change and their outcomes—e.g., Fukushima [42,43,44], financial crises—e.g., the 2008 Lehman Brothers bankruptcy [45,46], and military conflicts such as the Russian–Ukrainian war [47,48] may cause major disruptions in supply chains and generate direct repercussions on the duration and modalities of food distribution. All this, together with inflation in raw materials and energy [38], in addition to producing multi-sectoral and/or trans-regional crises, lead to major changes in the consumption habits of affected populations [49]. This impact, paradoxically positive from the point of view of combating the FLW phenomenon, manifests itself in the form of an “adjustment game-changer” of food purchase, preparation, and consumption practices [48,49], but also new skills (IT, in particular—[50,51]), adoption of healthy eating routines (either in the form of diets or by implementing a mealtime programme with the active involvement of family members and by buying quality, more expensive but nutritionally valuable food), as well as increased creativity and cooking abilities [31,32,52].

Moreover, given the sharp increase in food prices recorded on international markets in the last two years versus the lower purchasing power of consumers, food waste is not only an environmental and financial issue, but also an ethical and/or social one [27]. It requires further research from multi-, pluri-, and interdisciplinary, i.e., cross-societal perspectives, since behaviour, motivations, attitudes and/or perceptions differ according to traditions, regions, backgrounds (rural and urban), geographical, and generational influences [31,38,53].

At the European level, an increased level of food waste has been identified, bringing into question the need for appropriate educational programmes on how to identify viable solutions to combat the issue, with programmes to be carried out particularly at the household level. It is recommended to restore their consumption behaviour by explaining the importance of proper planning of food purchases by diversifying their ways of cooking and storing uneaten food [43,53,54].

The literature indicates that cooked and/or purchased food at the household level has often partially resulted in waste. To counter this behaviour and provide better awareness related to proper food consumption [55], the regional and local authorities, as well as other institutional actors, should educate citizens to avoid food loss and waste. Furthermore, alternative ways of consuming food which have the risk of becoming waste are being identified. Direct results translate into a visibly lower percentage of wastage of raw materials and processed ingredients [38,53].

Similarly, also encouraged by authorities and their direct collaborators are the introduction of shopping lists, but also predefined menus, individual or family diaries, as well as the food stocks’ inspection within households (pantry, cellar, and/or fridge) before shopping, are also optimal ways of moving from a compulsive, irrational purchase behaviour to a sustainable one, with consequences on the food waste management [24,31].

Consumption patterns, along with food purchasing habits [56,57], have become more sustainable during the recent sanitary crisis of the COVID-19 pandemic [58,59,60,61,62], with a positive impact on food waste management directly seen in shopping and cooking behaviours. First, the consumer’s responsibility increased in regard to the purchase of fresh vegetables and meat. During the sanitary crisis, consumers preferred to buy more qualitative food even though it was more expensive, and threw away less, with preference of local producers as a way of showing community support. Digitalization brought visible change in the perspective of consumers towards new ways of purchase; lockdowns encouraged online shopping and the implementation of the e-platforms, and proximity food delivery techniques also boomed during the COVID-19 pandemic [19,21,63,64]. Food waste decreased, with consumers discovering new ways to recover food leftovers [30].

#### 2.1.2. Consumer Clusters in FLW Literature

We noted several ways of population segmenting (cluster identification). The eco-friendly attitude and anti-waste behaviour are evidenced in the case of composting waste right in the household, with consumer segments going from average composters, disinterested city dwellers, helpless apartment dwellers, and active environmentalists [65]. Awareness of the effects of food waste is supported precisely by policies promoting home composting, especially among young people and families with children.

Depending on how citizens perceive food waste, a distinction is made between non-wasters—those who waste food and treat waste recklessly (people in rural and small towns with relatively low levels of education and who do not think waste is a problem in itself); the cautious—those responsible for not throwing away food (especially educated people from urban areas, because all actors in the agri-food chain are partly responsible); and the ignorant—the highly educated ones, eco-responsible since childhood or adolescence (people from urban areas, with healthy habits, aware of their role in society and who assume to share FLW responsibility with the other actors in the agri-food chain) [31,32]. Wasters, careless, and careful consumers have not yet improved their attitudes sufficiently, which requires further action to raise awareness of behavioural developments [66,67] in times of transition, as well as the organisation of broader campaigns to educate consumers and develop a mindset in favour of reducing food waste, i.e., understanding the related challenges and, especially, the consequences they have.

The literature has also considered clustering consumers regarding their food waste behaviour, thus identifying relevant waster typologies depending on socio-demographic characteristics, as well as on their food eating behaviour and the amount of food that they throw away. For instance, depending on consumers’ origin from four different European geographical areas, age, gender, and eating history, the literature [38] shows that both households and final consumers, depending on the food they consume (evaluated according to the degree of necessity, cost, nutritional importance, mode of consumption, quantity), can be clustered according to their habits and eating behaviours. Consumers’ socio-economic background and their socio-educational characteristics represent the control variables [38,63], with eating behaviours being largely affected by proximity contacts (people/groups with whom consumers interact regularly) and established long-term relations.

From a socio-economic perspective, the following five consumer clusters, depending on their food waste behaviour, could be delimited [68]: conservative, self-indulgent, indifferent, consumerist, and eco-responsible. Depending on their household income levels, the literature [39] identified three broad categories of consumer typologies, including unaware, unaware but not wasters, and aware. In the current socio-demographic and economic framework, we note the geographical dimension, a variable in which one can observe three clusters [69] among Italians (EU country, South-Western Europe), according to the effects of food wastage by observing the price paid for food as well as the quantities normally purchased (clusters 1–3).

Foods with a high unit cost have a lower impact on generating food waste, while those with a low unit cost are thrown away in higher quantities, regardless of their perishability, with consumer segmentation also related to the use of food in the kitchen as secondary ingredients, reused in the preparation of other menus [69]. The regions of southern Italy show a significantly lower incidence of food waste than those in the centre or north of the country [70]. For example, consumers in Southern Italy place more emphasis on the quality of products, bought less frequently but at a higher price, which they nevertheless consume in full, reducing the resulting amount of food waste to zero. In fact, there are several clusters of consumers, as follows: “green wasters” are very aware of the problem of food waste because food waste is thrown away, and therefore, society must identify solutions to recover it or eliminate its effects; “red wasters” have a relatively low view of the environment and sustainability, and waste a small amount of food; and “blue wasters” are aware of the global FLW phenomenon and always act according to strict theoretical guidelines and their previous experience [38].

In addition to price awareness, household size, packaging format, and marketing also seem to impact the level of food waste [71]. From this perspective, research conducted on UK households tested the relationships between the frequency of purchase of semi-prepared menus and household food waste [72], delineating five profiles including epicureans, traditional consumers, occasional consumers, food retailers, and kitchen avoiders. Occasional consumers and food avoiders are addicted to semi-prepared menus in accordance with their lifestyle, falling into the typology of people who show a “buy more, waste more” behaviour [72].

The literature [73] also highlights consumers’ assiduous concern for environmental protection and ethical approach to food waste and loss, highlighting the existence of consumer typologies concerned about the amount of food waste polluting the environment, which include committed environmentalists, traditional, casual, and non-environmentalists.

The attitude of consumers, according to their age, can represent a segmentation coefficient [74,75]. According to the geographical area they come from, but also the traditions and/or habits practiced, families can also be classified according to their sense of guilt towards food waste [76] as follows: guilty wasters, caring wasters, “in denial” wasters, and saints. Interestingly, we found that there are wasteful shoppers who do not feel bothered by buying too much food, even if they fail to consume it, and “in-denial” wasters who always buy food they do not use but deny their way of acting. Similar research [45] highlights the existence of the following three clusters: the wasters, the cautious, and the virtuous who are aware of the importance of their involvement in the FLW phenomenon but fail to identify their role, and therefore, successfully translate theory into practice.

### 2.2. Research Methodology

#### 2.2.1. Research Context

The food loss and waste issues are a major concern on the European Union’s agenda. D’Angelo [77] has made a foray into the EU’s efforts over time to identify solutions to counter FLW—these developments are contained in various reports, documents, and procedures, such as the 2015 “EU action plan for the Circular Economy”, the 2016 ”FUSIONS” Report, “The European Green Deal”, the “EU Farm to Fork Strategy”, or the “EU Platform on Food Losses and Food Waste”, which bring together stakeholders and key institution representatives. All of the above seek out measures to fight FLW, to disseminate best practices, and to assess progress made by EU Member States over time regarding this phenomenon [63,78,79].

The quantification of the FLW at the European level is, according to some authors [9,80,81], an extremely important milestone, reflecting the progress made by the EU in raising awareness of the importance of the issue. Currently, this quantification is carried out by Eurostat [36,37], which recently published the first monitoring report on the amount of food lost and wasted in the EU across the entire agri-food chain for the year of 2020. This report states that the total annual amount of food wasted was 127 kg/per capita, of which 70 kg was at household level. It can thus be seen that household wastage in 2020 represents about 55% of the total, with the rest of the amount being distributed as follows: 11% at primary production level, 18% at processing level, 9% at restaurant and other food service levels, while the distribution and retail chain-link accounts for only 7% [36]. Analysing the aforementioned statistics, one can realise that there is a lack of data on the amount of food lost and wasted in four of the EU Member States, namely, Belgium, Malta, Latvia, and Romania. Additionally, according to the same report, the highest amount of food wasted at the household level (kg/capita) is recorded in countries such as Portugal (124 kg/capita), Italy (107 kg/capita), and Luxembourg (91 kg/capita), with Bulgaria (26 kg/capita), Spain (30 kg/capita), and Slovenia (36 kg/capita) at the other end of the scale. The Eurostat report [36,37] draws attention to the impact of the COVID-19 health crisis on these results, knowing that the data were collected in 2020. Other researchers [19,82,83] noted an improvement in purchasing habits and anti-waste behaviour observed during the lockdown period, whereas consumers improved their practices to reduce daily waste.

#### 2.2.2. Research Design

This research aims to explore behavioural patterns of food waste in an emerging market using sociological survey techniques. This study uses a questionnaire administered through the CATI method to delimit different typologies of consumers according to the way they contribute to the reduction, promotion/distribution of food waste. This research seeks to identify various behavioural typologies regarding food consumption that favour or do not favour waste.

This study used specific steps outlined in [84], including the design and development of the research instrument based on the literature, the application of the research instrument, data collection, and data analysis.

The inclusion criteria for participants in this study are individuals who live in the emerging market under investigation and who are over the age of 18. The exclusion criteria are individuals who do not meet the age criteria and those who are not willing to participate in the study. The premise underlying the evaluation of these concepts lies in the need to find out whether food wastage is more dependent on external factors or more influenced by people’s awareness of the strategic role of wastage in their development and progress. Understanding these behavioural patterns can help companies as well as society to counteract the adverse effects of food waste, to educate consumers, and to identify the levers needed to reduce the impact of this phenomenon and its downsides.

**Figure 1:**
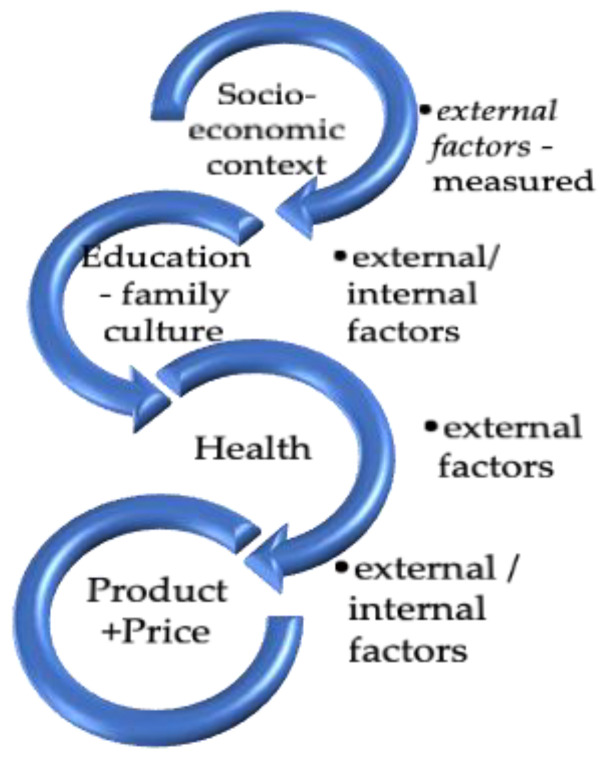
Instruments of research—profiles/patterns of food waste.

The survey instrument used a multidimensional approach that integrated various perspectives of researchers who studied the topic (refer to Figure 1). The questionnaire incorporated several elements that measured relevant concepts related to the investigation of food wastage. These included possible causes of food wastage, socio-economic-political factors that promote food wastage, properties of food products [38,48], consumers’ behavioural skills on food management [31,53], food price sensitivity [45,46], and personal care for purchased food [43].

During the empirical testing of the model, the aim was to elucidate the factors that could define the phenomenon of food waste, as outlined in Table 1 (refer to indicators/factors). To measure these factors, all items were assessed using a Likert-type scale with five response options, ranging from “1—totally disagree” to “5—totally agree”.

The study collected data from a sample of 1742 participants who were 18 years old or older. The sampling method used was simple random sampling, with a maximum allowable error of ± 2.39%. The data were collected via computer-assisted telephone interviewing (CATI) during the period of 17–28 March 2022. This survey was part of the internal research programme of the Romanian Institute for Evaluation and Strategy (IRES), which was self-funded.

#### 2.2.3. Hypothesis Development and Research Procedures

The responses collected in this study were analysed using econometric and mathematical models. To test the research hypotheses, statistical analysis was conducted using SPSS 17.0 software. As recommended by the literature [85,86,87], different analyses were performed, such as descriptive statistics, but also the reliability and the validity analysis, factor analysis, and cluster analysis. The analyses began with a test of marginal homogeneity, followed by reliability tests to ensure that the constructs in the survey were valid. Fleiss’s kappa hypothesis was used to test the agreement among respondents (H_1_), factor reliability (H_2_), and the discriminating power of the research instrument (H_3_–H_7_) to identify different types of food waste behaviour [85,86,87]. The goal was to identify specific factors and dimensions in the data through factor analysis using Varimax rotation. The factor components were selected based on a reliability value of at least 0.6 [85,86,87]. Factor reliability was confirmed using item-to-total correlation and Cronbach alpha [85,86,87].

**Figure 2:**
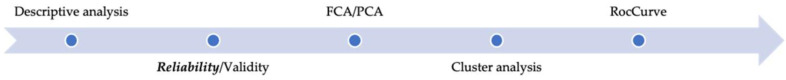
Research instrument—structure created according to [85,87,89].

With the help of the principal component analysis (PCA), representative dimensions were delimited. Here, the observed variables were grouped, while multicollinearity was tested so that the variable space could be transformed into an optimal one [87]. The Kaiser–Meyer–Olkin test was performed to ensure the reliability of the factor analysis [85,86,87]. Finally, cluster analysis was performed to identify food waste typologies (see Figure 2), and the discriminatory power of the research instrument was confirmed using the ROC curve [88,89]. The ROC curve allows the assessment of the predictive power for the model, to identify different types of food behaviours and profiles that were either focused or unfocused on food waste. The results show that the research instrument was reliable and discriminatory. The hypotheses are as follows:

**Hypothesis** **1** **(H_1_):** *Food wastage is more dependent on external factors than on individuals’ awareness of the strategic role of waste in their development and progress.*

**Hypothesis** **2** **(H_2_):** *Individuals’ awareness of the strategic role of waste in their development and progress has a greater influence on food wastage than external factors.*

**Hypothesis** **3** **(H_3_):** *The research instrument (questionnaire) can effectively measure the extent to which individuals’ awareness of the strategic role of waste influences food wastage.*

**Hypothesis** **4** **(H_4_):** *These variables are used to identify representative factors that generate awareness of the strategic role of waste in its development and progress.*

**Hypothesis** **5** **(H_5_):** *Factors that help individuals to understand the strategic role of waste in their development and progress can be grouped into one or more dimensions.*

**Hypothesis** **6** **(H_6_):** *There are statistical differences in the agreement on the measured characteristics that contribute to the awareness of individuals about the strategic role of waste in their development and progress (the proportion of weak towards non-existent concords is not equal to the one of total agreement).*

**Hypothesis** **7** **(H_7_):** *The research instrument can effectively discriminate between individuals with high and low levels of awareness of the strategic role of waste in their development and progress in relation to their food wastage behaviours.*

#### 2.2.4. Sample Description and Socio-Demographic Profile of Respondents

The data were collected through a CATI-based random sample survey, with a maximum tolerated error of ± 2.39%. The survey sample comprised 1742 individuals aged 18 years and above with 5 socio-demographic features, including gender (49% male and 51% female), age (18–65 + split as follows: 26% aged 18–35, 29% aged 36–50, 24% aged 51–65, and 21% aged 65+), education level (37% primary, 48% secondary, and 15% higher education), place of residence (54% urban and 46% rural), and region of origin (34% from Transylvania–Banat, 45% from South Bucharest–Dobrogea, and 21% from Moldova, as presented in Table 2).

To measure the degree of acceptance of the variables considered in the model designed to identify food behaviour types and profiles focused or unfocused on food waste, centrality indicators were calculated through descriptive analysis. These included standard deviation (SD), minimal quantity thought one, and maximal thought five indicators of normal skewness and kurtosis of data, which are recommended as valuable indicators for measuring variable acceptance [90,91]. A higher index value indicates greater respondent acceptance of the model’s indicators.

Respondents’ perceptions of the model items were analysed, revealing that 23.4% expressed full agreement with the questionnaire items, while 48.1% expressed partial agreement. Crosstab analysis was applied in the descriptive analysis stage to determine food purchasing behaviour during the pandemic crisis. Results show that 50% of respondents prefer purchasing from supermarkets, 17% from hypermarkets, 15.6% from neighbourhood shops, 5.4% from food markets, 9.0% from their own household or from the countryside, 1.8% from other sources, and 0.1% from online sources.

Respondents reported adopting stock-building behaviour for basic products such as flour, yeast, tinned food, oil, medicines, water, cleaning products, and hygienic paper during times of crisis. These items were purchased from shops they were familiar with, with priority given to supermarkets, hypermarkets, and neighbourhood shops, followed by food markets, their own household or countryside, local/regional producers, and online sources.

## 3. Results

### 3.1. Model Consistency

The analysis proceeded with various tests, including an agreement test, percentage agreement, Fleiss’s kappa, Cohen’s kappa correlations, and intraclass correlation. H_1_ presumed the agreement between the factor ratings as a reliable basis for the food behaviour model, which allows the identification of food waste behavioural profiles. The results based on the kappa analysis (kappa = 0.373, Z = 68.163, *p* = 0.001) show moderate agreement on the food behaviour factors/indicators to identify the food waste behavioural profiles (see Table 1, factor column), which accepts H_1_.

The test also showed sensitivity to the measured characteristics, with Cronbach’s alpha = 0.883 > 0.7, Mean = 19.16, and Std. Dv. = 5.578. Thus, the research instrument used was adequate for the purpose for which it was constructed, indicating that the test is unidimensional. The discrimination coefficient values are adequate. With a value of 0.883 (95% CI (0.849, 0.903)), the Cronbach alpha indicates that the participants’ assessment is consistent; they recognise the reliability of the factors within the proposed model. With a value of 0.424 (95% CI (0.409, 0.471)), the Single Measures test shows a moderate influence [92], validating H_2_.

In the next step, the differences between the groups were examined, and the interaction effects within the survey items were computed [85,86,87]. Therefore, a one-sided ANOVA testing was computed (F = 15.282, *p* = 0.000). It highlighted significant differences between the measured variables, as evidenced by the divergent responses of the participants. Thus, it can be inferred that the instrument used to measure food waste behaviour profiling is effective. Applying Hotelling’s T-square test (F = 182.340, *p* = 0.001) showed that there is discriminatory power. This indicates that the means of the analysed items are distinct, and therefore, it can be concluded that there are variations among the respondents’ answers [85,86,87], thus confirming the validity of H_3_. In summary, the questionnaire designed to delineate dietary behaviour based on food waste is well-suited for the research purpose.

The questionnaire used to measure dietary behaviour in relation to food waste was validated using a factor analysis. This method assumes that a few basic structures are responsible for causing correlations between many observed variables [85,86,87]. To identify these latent constructs, the measurement model needs to have a reliability value of at least 0.6 [85,86,87]. Therefore, the factor analysis allowed the transformation of the variable space [87]. The Kaiser–Meyer–Olkin test was conducted first, and a value of 0.872 > 0.50 and a *p*-value of < 0.001 was obtained, indicating that it was appropriate to use a factor analysis [93,94].

The results show that the questionnaire used was highly consistent and appropriate for investigating the phenomenon of interest. The sample size was sufficient, and the variability of the data was caused by the instrument generated. Bartlett’s test of sphericity was used to test the correlation between the variables, and the result (15988.566, Sig = 0.000) rejected the hypothesis that the variables/items were not correlated (H_4_). To identify the behavioural profiles related to food waste, seven principal components were selected, explaining 54.321% of the variance, each with Eigenvalues ≥ 1 [85]. Although the rotation procedure was applied, the number of dimensions could not be reduced; the variance also remained unchanged. Therefore, hypothesis H_5_ was accepted.

To test H_6_, the respondents’ perceptions of the measured attributes were checked to identify clusters of behaviours related to food waste. The chi-square test was used to determine if the proportion of poor agreement versus no agreement was not equal to the total agreement. According to the test, the alternative hypothesis was accepted, and the null hypothesis was rejected. The result of the test showed significant differences between agreement and disagreement on the acceptance/disagreement of the items used to identify the behavioural profiles towards food waste. The result was not due to random sampling variation, indicating that the respondents did not guess the answers [85,86,87].

Next, the analysis continued with Hierarchical and K-means clustering. This allowed us to identify clusters of respondents who relate similarly to the phenomenon of food waste. Three clusters were obtained using the K-means method, and the relationship between the resulting clusters and food waste behaviour was assessed using the chi-square test. The clusters were delineated based on attributes such as purchasing products at the lowest price, preparing food at home, serving food in restaurants, ordering food, age, respondents’ concern about the price paid for food that is thrown away, products being thrown away because they are not stored, repackaging products, not eating food according to its expiry date, storing basic foods such as flour, toilet paper, etc., financial crisis, food crisis, etc. The collected inertia values significantly exceeded the intra-cluster inertia values, indicating that the factors allowing the delineation of behavioural profiles on waste are uni- or multidimensional. Ward’s method was applied on the principal components’ score to determine the number of clusters, and the agglomeration schedule was checked with the maximization of the Square Euclidian distance. Statistical significance was used for *p* < 0.05 [85,89]. Seven factors were identified that contribute to food waste behaviour, including (1) attitudes towards food waste, (2) household income, (3) food storage habits, (4) food safety concerns, (5) meal planning and preparation, (6) purchase behaviour, and (7) socio-demographic characteristics.

Attitudes towards food waste refer to how individuals perceive the problem of food waste and whether they take responsibility for their own contribution to the issue. Those who have a high level of concern for food waste tend to be more mindful of their consumption and waste habits. This factor is an important predictor of food waste behaviour as it influences the individual’s decision-making process when it comes to food consumption. Household income also plays a significant role in food waste behaviour. Individuals with higher incomes tend to waste more food than those with lower incomes. This could be due to a variety of reasons, such as over-purchasing, lack of meal planning, and a general lack of concern for the cost of food waste.

Food storage habits refer to the methods individuals use to store and preserve their food. Proper food storage can help to reduce food waste by extending the shelf life of products. Individuals who have good food storage habits tend to waste less food than those who do not. Food safety concerns refer to individuals’ level of awareness and concern about the safety of their food. Those who have a high level of concern about food safety tend to be more cautious about the expiration dates and storage of their food. This can lead to less food waste as they are more likely to consume products before they expire.

Meal planning and preparation are also important factors in food waste behaviour. Individuals who plan their meals in advance tend to waste less food as they can buy and prepare only the amount of food they need. Additionally, people who are skilled in meal preparation tend to waste less food as they can reuse leftover ingredients. Purchase behaviour refers to the purchasing habits of individuals, including the types and quantities of food they purchase. Those who buy in bulk or at a discount tend to waste more food than those who purchase only what they need. Additionally, people who purchase convenience items such as pre-cut fruits and vegetables are more likely to waste food. Age, gender, and education level can also influence food waste behaviour. For example, Millennials or Generation Z tend to waste more food than Gen Xers or Baby Boomers. People with higher education levels tend to waste less food than those with lower education levels.

### 3.2. Cluster Description

**Figure 3:**
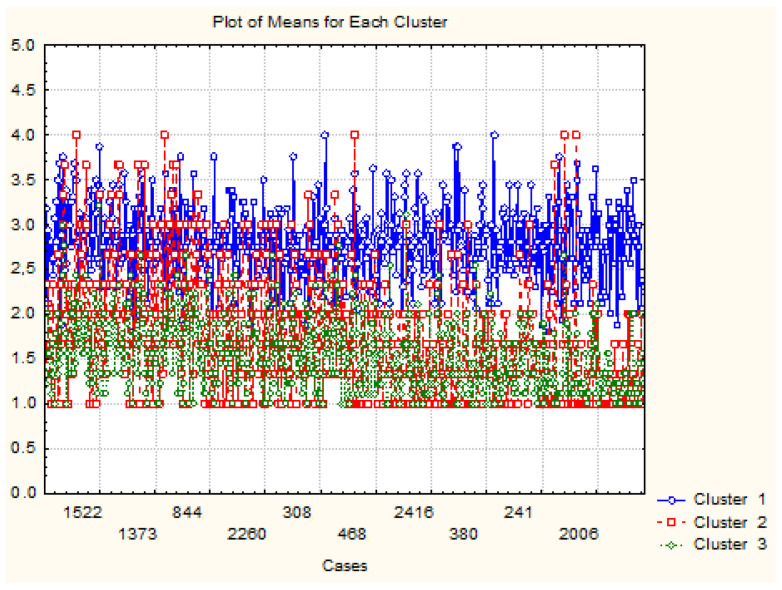
Cluster analysis.

In the study, we identified three consumer typologies (see Table 3 and Figure 3) based on their food waste behaviour, including (1) low-income young wasters, (2) conscious middle-age wasters, and (3) well-educated mature non-wasters. These typologies can be used by policymakers and marketers to develop targeted strategies to reduce food waste. A description of each cluster is also presented in Table 3.

#### 3.2.1. Cluster 1—Low-Income Young Wasters

Cluster 1 gathers 761 respondents, with an average age of 18–35 and an average education level. These consumers have a net household income of up to RON 2000 (about EUR 400) and typically throw away 3–4 kg of food per month. They prioritize buying local products and rely heavily on shopping lists when they go shopping, often basing their decisions on food sales or special offers. Price and origin are important factors in their food choices, with a preference for local products. They do not pay much attention to the sensory characteristics of the food they buy and do not buy in large quantities or stockpile food due to a lack of storage space. The expiry date is not a significant criterion when choosing which foods to consume. The leftovers are usually thrown away instead of being saved for another meal. These consumers cook at home frequently and rarely eat out, instead opting for regular takeout. During the pandemic, they moderately stocked up on essential products such as flour, tinned food, oil, cleaning products, toilet paper, and yeast. They report a slight increase in the quantities of water, oil, medicine, and fuel bought in the last month. They express concern about various crises, particularly financial and energy ones, but also towards armed conflicts.

Understanding these underlying factors can help policymakers and food industry stakeholders develop targeted interventions and education campaigns to reduce food waste among this group. Members of this cluster may have a lack of knowledge about how to properly store and use food, leading to spoilage and waste. In this cluster, consumers prioritize buying local products, which may contribute to their food waste habits. One possible explanation for this is that local products may have a shorter shelf life than products that have been transported from farther away. 

#### 3.2.2. Cluster 2—Conscious Middle-Age Wasters

This cluster consists of 560 respondents, comprising conscious middle-age wasters who are in cognitive dissonance between their overt and actual behaviour. These consumers are educated, mature, and have a higher income, with an age range of 36–50 and higher education levels. They have a net household income of over RON 2000 (EUR 400) and throw away more than 4 kg of food per month. They moderately use shopping lists when they go shopping and show moderate importance to promotions. For them, the price of the product is important in the buying process, but they prefer quality food such as organic and local products. They buy only what they need and claim to be neutral to concerned about the amount of food they throw away. They do not focus their shopping only on in-store promotions or special offers and do not buy in large quantities. They do not store or repackage food and do not consider the expiry date of products. They throw away food because it is burnt or not considered tasty by some family members. They pay moderate attention to the food smell and state that they cook moderately at home, they eat often in restaurants, and sometimes order food, paying high attention to the food they eat. During the pandemic, they moderately stocked up on flour, canned food, oil, and toilet paper, and minimally on medicines, cleaning products, and water. They report a slight increase to not at all in the amount of oil, medicine, and fuel bought in the last month, but not food. They are aware of all types of crises (sanitary, armed conflicts, political, etc.), particularly financial ones.

In Cluster 2, the conscious middle-age wasters, their behaviours may be due to a cognitive dissonance between their stated values of reducing food waste and their actual behaviours. By observing the consumers in this cluster, the authors could delve into their attitudes towards food waste and how they relate to other values and priorities. For example, these consumers may prioritize buying high-quality, organic, or locally sourced products, but then struggle to use them up before they spoil. So, consumers may experience cognitive dissonance because they value quality food and want to support sustainable and ethical food practices but may struggle with implementing these values in their day-to-day lives due to factors such as convenience and affordability.

#### 3.2.3. Cluster 3—Well-Educated Mature Non-Wasters

This cluster consists of 425 respondents, comprising well-educated mature non-wasters, with an age over 50 and higher education levels. These consumers have a net household income over RON 4000 (EUR 800) and throw away up to 1 kg of food. They consider the shopping list necessary, but not vital, and attach little importance to food sales in shaping their food purchase decisions. The price of food has very little importance in their buying process, and they consider local food important, but are not focused on it alone. They are very careful about the smell of the food they buy and express concern about the amount of food they throw away. They buy in large quantities, store food, and are mindful of product expiry dates, rarely throwing food away. They express moderate concern about all types of crises (sanitary, armed conflicts, etc.) but are very worried about the possible consequences of a financial and energy crisis. During the pandemic, they minimally to moderately stocked up on medicines and fuel, but not food. They declare that they cook moderately to rarely and prefer to order and eat in restaurants. In Cluster 3, the well-educated mature non-wasters, their behaviour may be due to a combination of factors such as their mindful approach to shopping, buying in bulk, and being careful of expiry dates. By observing the Cluster 3 consumers who take a mindful approach to food waste, the authors could explore the motivations behind this behaviour. One possibility is that the higher education and income levels have increased their awareness of the environmental and economic impacts of food waste. They may also have more resources available to them, such as larger kitchens and storage spaces that make it easier to buy and store food in bulk. So, the authors could explore whether the consumers are driven primarily by environmental concerns, or if other factors, such as health or financial considerations, play a role. Additionally, the authors could explore whether this group has any unique strategies for reducing waste that could be shared with the other clusters.

The ROC curve is a measure of how well a test can distinguish between individuals who engage in food waste avoidance behaviour and those who do not. The results show that the surface under the curve was significantly different from the curve area 0.5 (*p*: 0.041), with an ASC of 0.837 and a 95% confidence interval of 0.813 to 0.884. Therefore, there is discriminant validity in the model, meaning it can accurately measure and classify food waste behaviours among individuals with distinguishable profiles of focused and non-focused behaviours. The authors demonstrated that this model can be applied to other studies and can correctly identify normal versus abnormal situations, regardless of the respondent. Therefore, H_7_ is accepted.

## 4. Discussion

In the literature, consumer clustering based on their food waste behaviour has been quite intensively explored. This involves identifying relevant typologies of wasters based on socio-demographic characteristics, food eating behaviour, and the amount of food they discard. For example, Amicarelli et al. [38] cluster households and final consumers based on their eating habits and behaviour, as well as socio-economic and socio-educational variables. Five consumer clusters based on their food waste behaviour were also pinpointed [68], while [69] observed three clusters among Italians based on the effects of food wastage, price paid, and quantities normally purchased.

Consumers’ concern for environmental protection and ethical approach to food waste and loss has been highlighted in the literature, with committed environmentalists, traditional, casual, and non-environmentalists identified as consumer typologies [73]. The third cluster identified in this study, consisting of Romanians who are well aware of the contribution of food waste to pollution, supports these findings. Age and geographical location can also serve as segmentation coefficients, and families can be classified based on their sense of guilt towards food waste, such as guilty wasters, caring wasters, “in denial” wasters, and saints [76,95].

Interestingly, there are wasteful shoppers who do not feel bothered by buying too much food, and “in-denial” wasters who always buy food they do not use but deny their wasteful behaviour [76]. Another study identified three clusters, including the wasters, the cautious, and the virtuous, with the latter being aware of the importance of combating food waste but struggling to identify their role [45]. These findings are similar to the results of the current study, with the first cluster containing wasters, the second containing conscious wasters who recognise their behaviour as wrong, and the third consisting of well-educated mature consumers who engage in food waste reduction campaigns and try to incorporate a food-waste-combating behaviour in their daily lives.

The findings of this study are consistent with previous research that suggests that income and education level are important determinants of food waste behaviour among consumers. Furthermore, both this study and previous research recommend customised interventions to address food waste among different consumer groups. The emphasis on the economic, social, and environmental impacts of food waste is also a recurring theme in previous studies [22]. Overall, the present study builds on and reinforces the existing research on food waste [5,8,96], highlighting the need for continued efforts to reduce waste and its associated negative impacts.

There are two main routes to food waste behaviour among consumers; these routes include waste due to lack of attention, and waste due to lack of intention [96]. The authors emphasize the importance of understanding these routes to design effective interventions to reduce food waste.

## 5. Conclusions

Food waste has significant economic, social, and environmental impacts. From an economic perspective, wasting food means wasting resources, revenues for producers and retailers, but also increasing the costs with its disposal. Socially, food waste generates food insecurity, as people worldwide lack access to nutritious food. Environmentally, food waste leads to greenhouse gas emissions, fostering climate change, and diminishing valuable natural resources such as water and land. Therefore, wasted food must be reduced in a common effort by all stakeholders, including the food industry, producers, retailers, and consumers. Potential solutions include improving the supply chain efficiency, reducing overproduction, educating consumers, and thus, changing their behaviour. Furthermore, food recovery programmes are to be implemented, such as developing and supporting food banks and supporting any food rescue initiatives, which can redirect food surplus to people in need.

While the theoretical findings of our research and evaluations already presented here are useful for the food supply initiatives and further investigations on similar topics, we consider that practical exercises and activities would be highly appreciated, such as multi-actor cooperation (i.e., NGO—university—caterer cooking contests using saved ingredients and food items) through educational campaigns in schools and universities, and the introduction of a new subject in the school curricula with the application of the already-existing textbook of sustainable development, “Respect for resources”, an initiative of the Food Waste Combat NGO in Romania currently at the stage of optional course in some of the Romanian schools [97].

Our results also show how the FLW behaviour has evolved in recent times due to the tremendous and impactful crises that have affected contemporary society. Crisis situations, such as the late COVID-19 pandemic (sanitary crises); the armed conflict between Russia and Ukraine, which has caused not only migrations, but has also dramatically affected the supply chains of raw materials and negatively impacted energy prices; but also financial crises, have largely affected all consumers. Consumers within the identified clusters relate differently to the crisis situations, also displaying a distinct behaviour towards FLW.

At the company level, we suggest a better communication between the food supply chain actors and the European and national public authorities taking into consideration good practices and successful campaigns already being undergone in developed countries involved in the FLW combat. While the reduction in food waste presents several challenges, it also presents an opportunity to improve economic, social, and environmental outcomes. Technological innovations such as smart packaging and tracking systems can help reduce waste by improving storage and transportation efficiency. Reducing food waste presents several challenges, including the need to educate consumers to change their food waste behaviour. The managers of companies should implement viable strategies and bring optimal solutions to consumers under the form of food consumption alternatives. By encouraging food banks, food waste can be diminished as well.

Among the limitations of this research, we can pinpoint the CATI method regarding the administration of the interviews. The CATI method relies on the willingness of respondents to participate in telephone interviews. This may lead to a limitation of the population coverage, as not everyone has a telephone number and not all respondents answer telephone calls. In addition, people who do not own telephones or who are unwilling to answer telephone calls may have characteristics or opinions that are different from those of the general population, and therefore may not be representative of the sample collected. At the same time, we found no significant correlations in the literature between the ownership of communication equipment and consumption or wasteful behaviours.

The CATI method can be disrupted by several factors that may limit the response, and therefore, the quality of the data collected. These factors include, for example, overly personal or intrusive questions, overly complicated or unclear questions, questions on sensitive or controversial topics, and so on. This criterion has been kept under control in the questionnaire validation process, as mentioned above.

A thorough analysis within the entire food supply chain and the participation of all key actors (such as farmers, processors, traders, retailers, caterers, distributors, consumers, and NGOs) is highly recommended for a synergistic approach. We consider that a combined methodology adding semi-structured interviews with open-ended, no-leading, and neutral questions (a qualitative research) would complement the quantitative method and allow a more detailed and efficient problem exploration. The study was carried out on consumers, without dividing them according to the literature into Millennials, Generation X, etc. Future studies in this field can eliminate these shortcomings. Moreover, based on our study, we cannot generalise our results. To generalise the results, we need more participants in our study, but at the same time, the ROC curve said that the tool is discriminatory, so we can reuse it to facilitate generalisation.

## Figures and Tables

**Table 1:** Results of the factor analysis.

Factors	Variables	Loading	EV%	% of var.
Factor 1: Habits leading to food waste	Too many purchase transactions with discounts and special offers	0.631	15.099%	13.964%
	No shopping lists	0.632		
	Products packaged in too large quantities	0.676		
	The need to make room in the fridge for new products	0.695		
	No proper storage for products	0.703		
	Unproper repackages for non-finished products	0.705		
	No priority to best-before food	0.724		
	Ignorance of expiry date	0.715		
	Food preparation failures	0.745		
	No proper leftover management	0.625		
	Picky eaters’ improper management	0.647		
Factor 2: Geo-political and health risks influencing food consumption behaviour	War	0.711	13.007%	25.721%
	Food crisis	0.801		
	Labour crisis	0.721		
	Health crisis	0.793		
	Financial crisis	0.771		
	Energy resources crisis	0.791		
	Fuel crisis	0.736		
Factor 3: Cognitive and sensorial prerequisites of food products	Expiry date awareness	0.692	8.263%	31.984%
	Aspect of the product	0.745		
	Product packaging	0.717		
	Smell of the product	0.714		
Factor 4: Family-induced food waste habits	Finishing menus that are on consumers’ plate	0.684	6.956%	37.315%
	Consuming meals within the family	0.659		
	Do not use food for purposes other than for eating	0.711		
	Do not throw away food leftovers and/or eat leftovers later (next day)	0.661		
Factor 5: Price sensitivity	Buy products on sale	0.822	6.277%	42.592%
	Buy products at the lowest possible price	0.774		
	The price is attraction #1	0.606		
Factor 6	How often do you eat ordered food	0.788	5.741%	51.240%
Factor 7 Conscious consumer behaviour	Use a shopping list	0.601	5.641%	54.321%
	Buy quality products (organic, local)	0.746		
	Buy only products that you need/plan to use	0.854		
	To what extent you and your family buy food	0.602		
	Pay attention to the producer	0.842		

Note: EV: Eigenvariance; % of var: percentage of variance. The factors were automatically extracted by SPSS in the established order of the software. Used extraction method: principal axis factoring. Used rotation: Varimax with Kaiser Normalization; rotation converged in 6 iterations.

**Table 2:** Socio-demographic profile of respondents.

Feature	%	Feature	%	Feature	%
Gender		Education		Geographical Region	
Male	49%	Primary	37%	Transylvania–Banat	34%
Female	51%	Secondary	48%		
**Age**		Higher	15%	Bucharest–Dobrogea	45%
18–35	26%	**Residence**			
36–50	29%	Rural	46%	Moldova	21%
51–65	24%	Urban	54%		
65+	21%				

**Table 3:** Description of clusters.

Feature	Cluster 1	Cluster 2	Cluster 3
Number of Consumers	761	560	425
Name of Cluster	Low-income young wasters	Conscious middle-age wasters	Well-educated mature non-wasters
Net Household Income	Up to RON 2000 (EUR 400)	Over RON 2000 (EUR 400)	Over RON 4000 (EUR 800)
Age	18–35	36–50	Over 50
Education	Average education level	Educated	Well-educated
Average Monthly Food Waste (kg)	3–4	>4	<1
Shopping List Use	Heavy use	Moderate use	Little use
Importance of Price	Important	Moderate importance	Very little
Importance of Origin	High preference	High preference	Important
Sensory Characteristics	Low priority	Moderate priority	Very high priority
Food Stockpiling	No stockpiling	No stockpiling	Large stockpiling
Expiry Date Consideration	Not significant	Not significant	Mindful consideration
Eating Out Frequency	Rarely	Often	Rarely
Pandemic Stockpiling	Moderately stocked	Moderately stocked	Minimally to moderately stocked
Concern about Crises	Concerned	Neutral to concerned	Moderate concern

## Data Availability

Data can be requested from the IRES (Romanian Institute for Evaluation and Strategy).
